# NADPH Oxidase 2-Derived Reactive Oxygen Species Mediate FFAs-Induced Dysfunction and Apoptosis of β-Cells via JNK, p38 MAPK and p53 Pathways

**DOI:** 10.1371/journal.pone.0015726

**Published:** 2010-12-29

**Authors:** Huiping Yuan, Xiaoyong Zhang, Xiuqing Huang, Yonggang Lu, Weiqing Tang, Yong Man, Shu Wang, Jianzhong Xi, Jian Li

**Affiliations:** 1 Department of Biomedical Engineering, College of Engineering, Peking University, Beijing, China; 2 Peking University Fifth School of Clinical Medicine, Beijing Hospital, Beijing, China; 3 Graduate School of Peking Union Medical College and Chinese Academy of Medical Sciences, Beijing, China; Pennington Biomedical Research Center, United States of America

## Abstract

Dysfunction of β-cell is one of major characteristics in the pathogenesis of type 2 diabetes. The combination of obesity and type 2 diabetes, characterized as ‘diabesity’, is associated with elevated plasma free fatty acids (FFAs). Oxidative stress has been implicated in the pathogenesis of FFA-induced β-cell dysfunction. However, molecular mechanisms linking between reactive oxygen species (ROS) and FFA-induced β-cell dysfunction and apoptosis are less clear. In the present study, we test the hypothesis that NOX2-derived ROS may play a critical role in dysfunction and apoptosis of β-cells induced by FFA. Our results show that palmitate and oleate (0.5 mmol/L, 48 h) induced JNK activation and AKT inhibition which resulted in decreased phosphorylation of FOXO1 following nuclear localization and the nucleocytoplasmic translocation of PDX-1, leading to the reducing of insulin and ultimately dysfunction of pancreatic NIT-1 cells. We also found that palmitate and oleate stimulated apoptosis of NIT-1 cells through p38MAPK, p53 and NF-κB pathway. More interestingly, our data suggest that suppression of NOX2 may restore FFA-induced dysfunction and apoptosis of NIT-1 cells. Our findings provide a new insight of the NOX2 as a potential new therapeutic target for preservation of β-cell mass and function.

## Introduction

Type 2 diabetes is a multifactorial disease including genetic and many other environmental factors. Dysfunction of β-cell is one of major characteristics in the pathogenesis of type 2 diabetes [1]. The prevalence of obesity in a modern society has increased dramatically over the past few years and has reached epidemic proportions. The combination of obesity and type 2 diabetes, characterized as ‘diabesity’, is associated with excessive release of fatty acids from the expanded adipose tissue mass, leading to elevated plasma free fatty acids (FFAs) [2].

Dysfunction of β-cell is induced by several molecules including glucose, FFA, and certain cytokines such as TNF-α [3]. Elevated plasma FFA levels, which are often accompanied by obesity, may play a causal role in β-cell dysfunction. It is reported that acute FFA exposure stimulates insulin secretion, while prolonged FFA exposure decreases glucose-stimulated insulin secretion (GSIS) [4], [5]. However, molecular mechanisms linking FFA to β-cell dysfunction remain poorly understood.

Oxidative stress has been implicated in the pathogenesis of FFA-induced β-cell dysfunction. It has been suggested that increased reactive oxygen species (ROS) levels are the important trigger for β-cell dysfunction. Under diabetic conditions, ROS are increased in many tissues and organs and cause various forms of tissue damage in patients with diabetes. It is considered that enhanced ROS generation may act as a link between FFA and β-cell dysfunction [6].

We recently found that suppression of NADPH oxidase 2 (NOX2) substantially restores glucose-induced dysfunction of pancreatic NIT-1 cells. Here, we demonstrate the critical role of NOX2-derived ROS in dysfunction of NIT-1 cells treated with palmitate or oleate. We show novel data that NOX2-derived ROS may promote FFA-induced dysfunction of β-cell through JNK pathway.

## Results

### Increased ROS generation in NIT-1 cells treated with palmitate and oleate is mainly derived by NOX2

**Figure 1:**
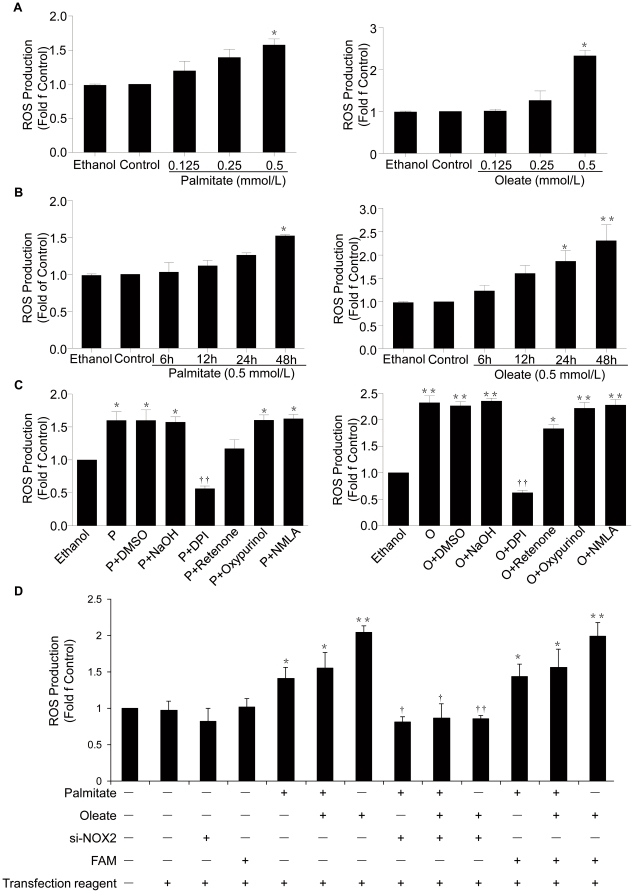
FFA-induced increased ROS generation in NIT-1 cells. ROS levels were dose- and time-dependently increased by exposure of NIT-1 cells to palmitate and oleate (A, B). The effects of different inhibitors of ROS-generating systems: DPI (NOX, 2.5 µmol/L), L-NAME (nitric oxide synthases, 50 µmol/L), Rotenone (mitochondrial respiratory chain, 1 µmol/L) and Oxypurinol (xanthine oxidase, 50 µmol/L) on palmitate- and oleate-induced ROS generation were analyzed (C). NIT-1 cells were transiently transfected with siRNA-NOX2 for 48 h followed by palmitate and oleate (0.5 mmol/L) treatment for 48 h, and ROS generation was measured (D). Data are present as mean ± S.E.M., n = 3 independent experiments. *p<0.05 and **p<0.01 by ANOVA test (Palmitate/Oleate *v.s.* control). †*p*<0.05 and ††*p*<0.01 by ANOVA test (siNOX2+Palmitate/Oleate *v.s.* Palmitate/Oleate).

To observe effects of FFA on ROS production in pancreatic β-cells, mouse pancreatic NIT-1 cells were treated with different concentrations of palmitate or oleate (0.15, 0.25, 0.5 mmol/L) for different time (6, 12, 24, 48 h). As shown in Fig. 1A and Fig. 1B, ROS levels were dose- and time-dependently increased by exposure of NIT-1 cells to palmitate and oleate. In order to further assess the source of ROS production, we investigated the effects of different inhibitors of ROS-generating systems: DPI (NOX, 2.5 µmol/L), L-NAME (nitric oxide synthases, 50 µmol/L), Rotenone (mitochondrial respiratory chain, 1 µmol/L) and Oxypurinol (xanthine oxidase, 50 µmol/L) on palmitate- and oleate-induced increased ROS levels. As shown in Fig. 1C, DPI significantly and Rotenone partially, but not L-NAME and Oxypurinol, inhibited generation of ROS in response to palmitate and oleate (0.5 mmol/L, 48 h). These results suggest NOX as a leading candidate for production of ROS in NIT-1 cells. Using RT-PCR, we have found expression of NOX2 and subunits such as p22^phox^, p67^phox^, p47^phox^ and Rac1—but not NOX1, NOX3, NOX4 and NOX5— in NIT-1 cells (data not show). To further assess the role of NOX2 in palmitate- and oleate-induced increased ROS generation in NIT-1 cells, we generated the siRNA targeting NOX2 mRNA (siRNA-NOX2) and transfected them into NIT-1 cells. The results indicate that transfection of siRNA-NOX2, but not control siRNA, significantly reduced ROS generation in NIT-1 cells treated with either palmitate or oleate (Fig. 1D), suggesting that NOX2 serves as the possible predominant source of ROS generation stimulated by palmitate and oleate.

### Treatment of palmitate and oleate induces dysfunction and apoptosis in NIT-1 cells

**Figure 2:**
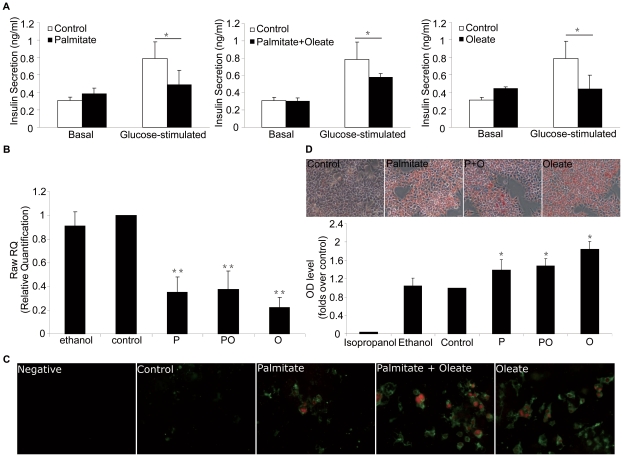
FFA- induced dysfunction and apoptosis of NIT-1 cells. Palmitate and oleate (0.5 mmol/L, 48 h) decreased glucose-stimulated insulin secretion (GSIS, 20 mmol/L D-glucose in KRBH) (A), reduced insulin mRNA level shown by real-time PCR (B), induced apoptosis assessed by double-staining with Annexin V and PI (C) and increased lipid accumulation detected by Oil Red O Staining in NIT-1 cells (D). Data are present as mean ± S.E.M., n = 3 independent experiments. *p<0.05 and **p<0.01 by ANOVA test (Palmitate/Oleate *v.s.* control).

Glucose-stimulated insulin secretion (GSIS) is the remarkable character of β-cell. To analyze the effect of palmitate and oleate on β-cell dysfunction, we determined the insulin expression and secretion in NIT-1 cells exposure to either 0.5 mmol/L palmitate or oleate for 48 h. ELISA showed unchanged basal (2.5 mmol/L glucose in KRBH) insulin secretion and decreased glucose-stimulated insulin secretion (GSIS; 20 mmol/L glucose in KRBH) in NIT-1 cells in response to palmitate and oleate (Fig. 2A). Moreover, mRNA levels of insulin were significantly reduced in NIT-1 cells treated with palmitate and oleate, as shown by real-time PCR (Fig. 2B), demonstrating that palmitate and oleate induce dysfunction of NIT-1 cells.

We next investigated whether apoptosis occurs in the NIT-1 cells treated with palmitate and oleate. Using Annexin V and PI kit, we found that NIT-1 cells treated with 0.5 mmol/L of palmitate and oleate for 24 h were double-stained, suggesting that palmitate and oleate induce apoptosis of NIT-1 cells (Fig. 2C).

Moreover, Oil Red O staining showed increased lipid accumulation in NIT-1 cells treated with palmitate and oleate (Fig. 2D).

### Palmitate and oleate impairs insulin synthesis in NIT-1 cells by PTEN-depend JNK activation and Akt inhibition

**Figure 3:**
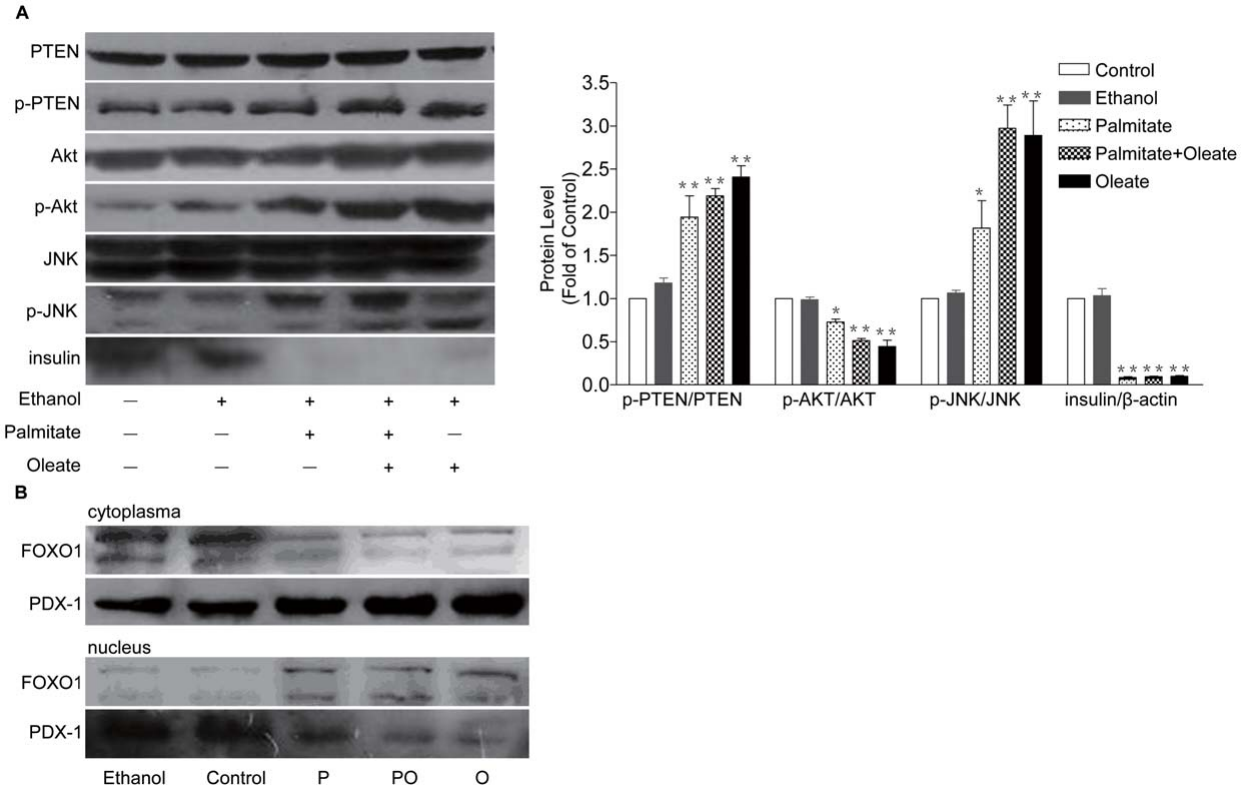
The signal transduction pathways involved in FFA-induced dysfunction of NIT-1 cells. Palmitate and oleate (0.5 mmol/L, 48 h) enhanced the phosphorylation of PTEN and JNK, and reduced the phosphorylation of Akt (A). FOXO1 content was impaired in cytoplasm but elevated in nucleus, while level of PDX-1 was increased in cytoplasm but decreased in nucleus (B). Data are present as mean ± S.E.M., n = 3 independent experiments. *p<0.05 and **p<0.01 by ANOVA test (Palmitate/Oleate *v.s.* control).

We then investigated what downstream pathways translate palmitate- and oleate-induced elevated ROS levels into dysfunction of pancreatic β-cells. Here, we focus on PTEN-depend JNK activation and Akt inhibition. As shown in Fig. 3, PTEN was activated in response to palmitate and oleate treatment in NIT-1 cells. In parallel with increased phosphorylation of PTEN, phosphorylation of the residue ^183^Thr/^185^Tyr in JNK was stimulated by palmitate and oleate treatment. Finally, exposure to palmitate and oleate reduced the phosphorylation of Akt. Western blot showed that FOXO1 content was decreased in cytoplasm but increased in nucleus. In contrast, level of PDX-1 was increased in cytoplasm but decreased in nucleus (Fig. 3B). Importantly, palmitate- and oleate-induced translocation of FOXO1 and PDX-1 led to impaired insulin synthesis in NIT-1 cells.

### Palmitate and oleate induces apoptosis in NIT-1 cells through p38MAPK and p53

**Figure 4:**
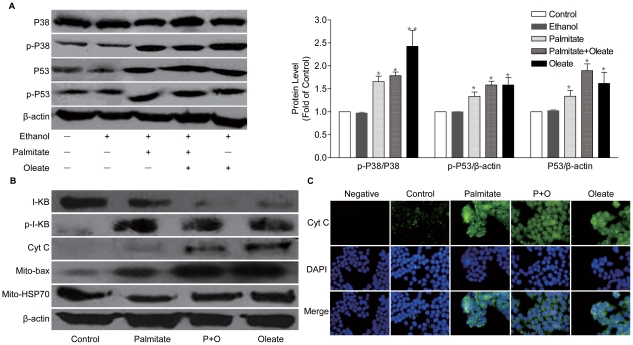
Molecular mechanisms involved in apoptosis of β-cells induced by FFA. Palmitate and oleate (0.5 mmol/L, 48 h) stimulated the phosphorylation of p53 and p38MAPK (A). Palmitate and oleate induced degradation of I-κB, phosphorylation of I-κB in ^32^Ser (B), translocation of Bax into mitochondrial (B), release and translocation of cytochrome C (B, C). Data are present as mean ± S.E.M., n = 3 independent experiments. *p<0.05 and **p<0.01 by ANOVA test (Palmitate/Oleate *v.s.* control).

We next sought to determine the mechanisms behind the apoptosis in β-cell induced by FFAs and to evaluate whether these factors correlated to the oxidative stress and apoptosis. As shown in Fig. 4A, exposure to palmitate and oleate for 48 h strongly stimulated phosphorylation of p38 MAPK and p53. Moreover, we found enhanced phosphorylation of I-κB in ^32^Ser, followed by degradation of I-κB, confirming NF-κB activation (Fig. 4B). The results suggest that NF-κB is also involved in the procedure of apoptosis of NIT-1 cells induced by palmitate and oleate.

We further assessed the molecules involved in FFA-induced apoptosis. Bax as a key factor in the process of apoptosis was measured by Western blot. Translocation of Bax into mitochondrial was found in NIT-1 cells treated with palmitate and oleate (Fig. 4B). Moreover, palmitate and oleate stimulated increased expression of cytochrome C and released it from mitochondrial to cytoplasm (Fig. 4B and 4C). These observations indicate that p38MAPK and p53 mediate the apoptosis of NIT-1 cells induced by palmitate and oleate.

### Suppression of NOX2 restores FFA-induced dysfunction and apoptosis of NIT-cells

**Figure 5:**
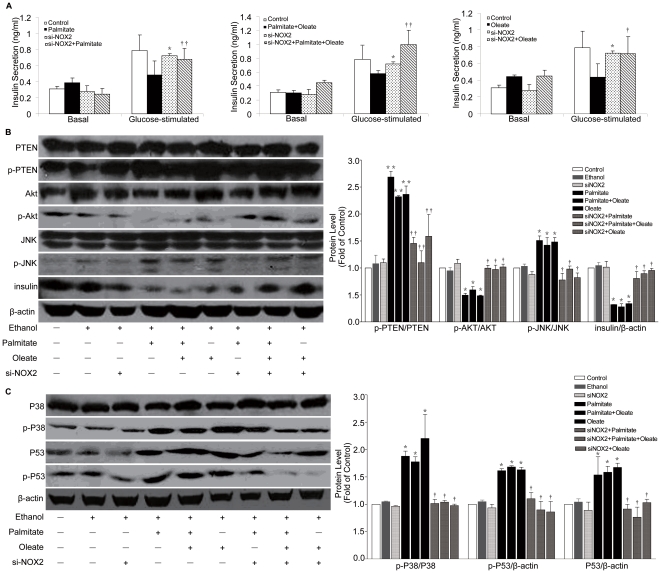
Suppression of NOX2 restores FFA-induced dysfunction and apoptosis of NIT-cells. NIT-1 cells were transiently transfected with siRNA-NOX2 for 48 h followed by palmitate and oleate (0.5 mmol/L) treatment for 48 h. The release of insulin (A), expression of insulin and activation of PTEN-JNK pathway (B), p38MAPK and p53 pathways (C) were measured. Data are present as mean ± S.E.M., n = 3 independent experiments. *p<0.05 and **p<0.01 by ANOVA test (Palmitate/Oleate *v.s.* control). †*p*<0.05 and ††*p*<0.01 by ANOVA test (siNOX2+Palmitate/Oleate *v.s.* Palmitate/Oleate).

To verify the key role of NOX2 in the dysfunction and apoptosis induced by palmitate and oleate, we transfected siRNA-NOX2 into NIT-1 cells. The results show unchanged basal (2.5 mmol/L glucose in KRBH) insulin secretion in NIT-1 cells transfected with siRNA-NOX2. However, NOX2 down-regulation reversed palmitate- and oleate-induced decreased glucose-stimulated insulin secretion (GSIS; 20 mmol/L glucose in KRBH) (Fig. 5A), increased JNK phosphorylation and decreased Akt phosphorylation in NIT-1 cells (Fig. 5B). Moreover, effects of palmitate and oleate on activation of p38MAPK and p53 were rescued via siRNA-mediated silencing of NOX2 (Fig. 5C). These results suggest that suppression of NOX2 may restore FFA-induced dysfunction and apoptosis of NIT-1 cells.

## Discussion

This study was designed to identify the mechanisms of lipotoxicity in diabetes and to test the hypothesis that NOX2-derived ROS may play a critical role in dysfunction and apoptosis of β-cells induced by FFAs. The underlying concept is that once the primary pathogenesis of diabetes is established, hyperglycemia and very commonly hyperlipidemia ensue and thereafter exert additional damaging or toxic effects on the β-cell [7]. It has been suggested that insulin resistance precedes development of type 2 diabetes. A common feature of insulin resistant states is high plasma FFA [8]. In addition to reducing insulin sensitivity in peripheral tissues, elevated plasma FFA levels may also contribute to the deterioration of β-cell function. A number of *in vitro* studies, using insulin-secreting cells and isolated islets, have attempted to identify the mechanisms of lipotoxicity in β-cell dysfunction. *In vitro*, prolonged exposure of isolated islets or insulin-secreting cells to elevated levels of FFAs is associated with inhibition of glucose-induced insulin secretion [9], [10] that has also been observed *in vivo* in rats [11] and humans [12], impairment of insulin gene expression [13]–[18] and induction of cell death by apoptosis [19], [20]. Accordingly, we stimulated pancreatic NIT-1 cells for 48 h with 0.5 mmol/L palmitate (a saturated fatty acid) and 0.5 mmol/L oleate (a -monounsaturated fatty acid). The results indicate that palmitate and oleate induced apoptosis of NIT-1 cells (Fig. 2C). Moreover, palmitate and oleate treatment decreased glucose-induced insulin secretion, impaired insulin gene expression, and increased lipid accumulation in NIT-1 cells, suggesting a state of β-cell dysfunction (Fig. 2A, 2B and 2D). Although it is reported that palmitate induced β-cell apoptosis whereas oleate protected NIT-1 cells from palmitate-induced lipoapoptosis [21], a plethora of studies has indicated that chronic exposure of β-cell to elevated levels of FFAs is accompanied by the loss of glucose-stimulated insulin secretion (GSIS) and a decrease in total insulin content [22]. Oleate has been shown to induce defects in GSIS in numerous pancreatic β-cell lines as well as rodent and human islets [23]. Recently, it has been suggested that chronic exposure of human pancreatic islets to oleate activates inflammatory and metabolic pathways that lead to oxidative stress, reduced β-cell insulin content, and inhibition of GSIS [2]. Moreover, the study in INS-1 demonstrated that exposure to oleate induced β-cell apoptosis, as did palmitate [24], [25]. Our results are consistent with these findings.

What is the molecular link between FFAs and β-cell dysfunction? In view of the current weigh of experimental evidence, it is now widely accepted that oxidative stress contribute to cell and tissue dysfunction and damage caused by lipotoxicity in diabetes. Recently, it has been also shown that chronic exposure to elevated fatty acids concentrations can damage different types of cells by a variety of mechanisms, but oxidative stress may be a common link in cell dysfunction [26], [27]. Increased FFAs metabolism may also lead to increased ROS production. It has been reported that FFAs initiates formation of ROS in pancreatic β-cells [28], [29]. Our results demonstrate that ROS levels were dose- and time-dependently enhanced by exposure of NIT-1 cells to palmitate and oleate (Fig. 1A and 1B). To further assess the source of ROS generation in response to stimulation of palmitate and oleate, we observed effects of different inhibitors of ROS-producing systems such as mitochondrial electron transport chain, NOX, nitric oxide synthase and xanthine oxidase. The results suggest that NOX may be the predominant source of ROS in NIT-1 cells, although Rotenone, a mitochondrial respiratory chain inhibitor, may partially inhibit generation of ROS in response to palmitate and oleate (Fig. 1C). The members of NOX family including NOX1, NOX2, NOX3, NOX4 and NOX5 have been identified in different tissues and cells [30]. Recently, it is reported that NOX2 and subunits such as p22^phox^, p47^phox^ and p67^phox^ are expressed in rat pancreatic islets [31]. We also found a similar expression pattern of NOX and partners in NIT-1 cells (data not shown). NOX2 down-regulation by transfection of siRNA-NOX2 reduced ROS generation (Fig. 1D), and, in turn, improved the function in NIT-1 cells treated with palmitate and oleate (Fig. 5), suggesting that NOX2 may be a leading candidate for production of ROS in NIT-1 cells. Recently, it was reported that NOX2 deficiency decreases β-cell destruction and preserves islet function in STZ-induced diabetes by reducing ROS production, immune response, and β-cell apoptosis [31]. Our results confirm the findings of *in vivo* study.

It is well known that excessive levels of ROS not only directly damage cells by oxidizing DNA, protein and lipid, but also indirectly damage cells by activating a variety of stress-sensitive intracellular signaling pathways such as NF-κB, p38MAPK and JNK/Akt [32], [33]. In oxidative stress state, PTEN is phoshorylated in ^380^Ser/^382/383^Thr, leading to activation of JNK by phosphorylation in ^183^Thr/^185^Tyr and decreased phosphorylation of Akt through increased PIP3 producing [34]. Activation of these pathways results in increased expression of numerous gene products that may cause apoptosis and play a major role in β-cell dysfunction.

The mechanisms underlying the decrease in glucose-stimulated insulin secretion by fatty acids are less clear, although recent studies have provided important insights into potential candidates such as G protein-coupled receptor GPR40 which may play a major role in the deleterious effects of FFAs on β-cell function, uncoupling protein-2 (UCP-2) which is suggested that UCP-2 modulates insulin secretion, and ATP-binding cassette transporter subfamily A member-1 (ABCA1) which impairs insulin secretion [7]. Further investigation is required to clarify the effects of these candidates on FFA-induced β-cell dysfunction. Regarding FFA-induced impairment of insulin gene expression in β-cell, we found that reduction of insulin gene expression in NIT-1 cells exposed to palmitate or oleate is accompanied by a decrease of PDX-1 expression in nuclei, suggesting the implication of PDX-1 in β-cell dysfunction. PDX-1, a homeodomain-containing transcription factor that plays a pivotal role in pancreatic development, β-cell differentiation, and in maintaining mature β-cell function, is reported to have a functional nuclear localization signal and undergoes nuclear translocation in response to several functional stimuli such as glucagon-like peptide-1 [35]. Conversely, in the present study, we show that PDX-1 also possesses a nuclear export signal in response to FFA. PDX-1 appears to be affected in its ability of translocation to the nucleus. Thus, it is likely that palmitate or oleate inhibition of insulin gene transcription is due to nucleocytoplasmic translocation of PDX-1 and decreased binding activity of PDX-1. More interestingly, palmitate- or oleate-induced nucleocytoplasmic translocation of PDX-1 is accompanied by a decrease in cytoplasm but an increase in nucleus of FOXO1 expression, suggesting that FOXO1 translocation might modulate the decrease of PDX-1 in nucleus. It bas been demonstrated that oxidative stress induces the nuclear translocation of FOXO1 through activation of the JNK pathway, which leads to the nucleocytoplasmic translocation of PDX-1 [35]. FOXO1 is reported to reduce PDX-1 promoter activity by competing with Foxa2 for DNA binding to the PDX-1 promoter [36]. However, function of FOXO1 in regulating intracellular localization of PDX-1 might not be due to a transcriptional event, because whole cell amount of PDX-1 was not altered by the modification of FOXO1 expression by adenovirus [35]. Further studies need to be performed to clarify the detailed mechanism underlying the FOXO1-induced nucleocytoplasmic translocation of PDX-1. We also found that phosphorylation of Akt was reduced in NIT-1 cells treated with palmitate or oleate, suggesting that Akt is involved in the FFA-mediated nuclear translocation of FOXO1. Moreover, our findings suggest the involvement of the JNK-Akt- FOXO1-PDX-1-insulin axis in the impairment of insulin expression, and this pathway may explain parts of the molecular mechanisms of β-cell dysfunction induced by FFAs. It has been reported that JNK activation is involved in the reduction of insulin gene expression by oxidative stress [37] and JNK activation induces the nucleocytoplasmic translocation of the pancreatic transcription factor PDX-1 and thereby reduces PDX-1 DNA binding activity [38]. In our findings, elevated phosphorylation of JNK is accompanied by reduced phosphorylation of Akt and FOXO1 in NIT-1 cells treated with palmitate or oleate. In conclusion, FFA-induced JNK activation and Akt inhibition resulted in decreased phosphorylation of FOXO1 following nuclear localization and the nucleocytoplasmic translocation of PDX-1, leading to the reducing of insulin and ultimately dysfunction of β-cells.

Several pathways have been proposed to mediate FFAs-induced apoptosis in β-cells, including p38MAPK, p53 and NF-κB pathways. It has been reported that p38 MAPK is activated by dual phosphorylation of ^180^Thr and ^182^Tyr residues, and p53 is activated by ^15^Ser residues. The phosphorylation of p38 MAPK and p53 has been widely used to represent its activation in response to oxidative stress. Our results suggest that p38MAPK and p53 mediated the apoptosis of NIT-1 cells induced by palmitate or oleate. Exposure of palmitate or oleate for 48 h strongly stimulated phosphorylation of p38MAPK and p53, accompanied by activation of NF-κB. NF-κB activation is pro-apoptotic in pancreatic β-cells. FFAs are able to activate NF-κB. NF-κB activation mediates expression of inflammatory genes, in term induces cell apoptosis. Moreover, It bas been suggested that NF-κB is involved in the procedure of apoptosis mediated by p53 [39].

In addition, JNK activation is known to induce cell apoptosis. Some transcription factors including c-Jun, ATF2, Elk1, c-myc, or p53 are major targets of JNK. Thus, JNK activation probably acts by modifying the expression of genes that play an important role in controlling cell death or survival. Therefore, the coordinate regulation of the genes controlled by JNK sensitizes β-cells to the proapoptotic action of FFAs [39].

Moreover, translocation of Bax into mitochondrial was found in NIT-1 cells treated with palmitate and oleate. Palmitate and oleate also increased expression of cytochrome C and released it from mitochondrial to cytoplasm. In addition, it has been shown that FFAs might contribute to β-cell apoptosis by decreasing insulin/Akt signaling [32], [33]. As a proapoptotic transcription factor, FOXO1 is also involved in β-cell apoptosis induced by FFAs [39]. More interestingly, our data suggest that suppression of NOX2 may restore FFA-induced dysfunction and apoptosis of NIT-1 cells. Our findings provide a new insight of the NOX2 as a potential new therapeutic target for preservation of β-cell mass and function.

## Materials and Methods

### Cell culture

NIT-1 cells derived from mouse pancreatic β-cells (ATCC, 20–30 passages) were cultured in low-glucose DMEM (5 mmol/L glucose, Gibco) supplements with 10% fetal bovine serum (Hyclone), 100 U/ml penicillin (Gibco) and 0.1 mg/ml streptomycin (Gibco) at 37°C in a humidified atmosphere of 95% O_2_, 5% CO_2_.

### Determination of ROS

Cells (3×10^5^ cells/ml) were incubated with 5 µmol/L of DCF-DA (Sigma) for 40 min at 37°C. The DCF fluorescence was measured by FACS with an excitation/emission wavelength of 488/525 nm.

### RNA isolation, RT-PCR and real-time PCR

Total RNA was isolated from NIT-1 cells using the Trizol Regent (Invitrogen). Reverse transcription (RT) was performed on 1 µg RNA at 60°C for 35 min using a Reverse Transcription kit (A3500, Promage) containing 0.5 µg random primers, 15 U AMV, 0.5 U RNasin RNase inhibitor. After reverse transcription, the cDNAs were used for semi-quantitative PCR using sets of specific primers as follows:

NOX1: 5′-GAAATTCTTGGGACTGCCTTGG-3′ and 5′-GCTGGAGAGAACAGAAGCGAGA-3′;

NOX2: 5′-AATCACTTTGCTGTGCACCTGA-3′ and 5′-AATTCCTTTTAGGGGGCCTGTGT-3′;

NOX3:5′AGCTGCCTTATGCCCTGTACCTC3′and 5′-AGGCCTTCAATAACGCGCCTCTGTC-3′;

NOX4: 5′-GGACGTCCTGGTGGAAACTT-3′ and 5′-GCAAACCCTTGGGTATTCTTTGG-3′;

P22 phox: 5′-GGAGCGATGTGGACAGAAGTA-3′ and 5′-GCACCGACAACAGGAAGTG-3′;

p47 phox: 5′-'CTATCTGGAGCCCCTTGACA-3′ and 5′-ACAGGGACATCTCGTCCTCTT-3′;

p67 phox: 5′-CCAGAAGACCTGGAATTTGTG-3′ and 5′-AAATGCCAACTTTCCCTTTACA-3′;

Rac-1: 5′-AGACAATTTGGGCACACCTC-3′ and 5′-GCTTCGTCA AACACTGTCTTG-3′;

β-actin: 5′-GTGGGGCGCCCCAGGCACCA-3′ and 5′-CTCCTTAATGTCACGCACGATTTC-3′.

An initial step of 94°C was used for 5 min followed by 30 cycles of 94°C for 1 min, 60°C for 1 min, and 72°C for 1 min. RT-PCR was finished with 72°C for 7 min. Fifteen micro liters of reaction were run on a 1.5% agarose gel and photographed on a UV transilluminator using a digital camera.

Real-time PCR was performed using A7500 Real-Time Thermal Cycler (ABI). The specific primers for real-time PCR are: 5′-CTTCTACACACCCAAGTCCCG-3′ and 5′-GTGCAGCATGATCCACAATG-3′ for insulin; 5′-CGTCCCGTAGACAAAATGGT-3′ and 5′-TTGATGGCAACAATCTCCAC-3′ for GAPDH. Amplification was carried out as recommended by the manufacturer: 25 µl reaction mixture contained 12.5 µl of SYBR Green PCR Master mix (Applied Biosystems), the appropriate primer concentration, and 1 µl of cDNA. The relative cDNA concentrations were established by a standard curve using sequential dilutions of corresponding PCR fragments. The data were normalized to GAPDH. The amplification program included the initial denaturation step at 95°C for 10 min, 40 cycles of denaturation at 95°C for 10 s, and annealing and extension at 60°C for 1 min. Fluorescence was measured at the end of each extension step. After amplification, melting curves were acquired and used to determine the specificity of PCR products.

### Determination of apoptosis

To determine apoptosis in INT-1cells, cells were double-stained with Annexin V and PI kit (Baosai) according to the manufacturer's protocol. Stained nuclei were immediately visualized by fluorescence microscopy.

### Oil Red O staining

After incubation in the presence or absence of palmitate or oleate for 48 h, confluent cell monolayer was fixed in phosphate-buffered formalin (10%) for 15 min, rinsed with water followed by 70% ethanol, and stained with Oil Red O solution (6 parts of saturated Oil Red O dye in isopropanol +4 parts of water) for 15 min. Excess stain was removed by washing with 70% ethanol. The stained cells were finally washed with water. The cell monolayer was then incubated for 5 min with 1.5 ml of 4% Nonidet P-40 in isopropanol which dissolved stained oil droplets. The absorbance of the dye-triglyceride complex was measured at 520 nm after suitable dilution.

### Immunofluorescence

The coverslips of NIT-1 cells were fixed for 10 min with ice-cold methonal at −20°C. The cells were incubated with polyclonal antibodies at 4°C overnight, and then were labeled with Fluorescein Isothiocyanate (FITC)-conjugated anti-rabbit IgG at 37°C for 60 min and stained with DAPI at room temperature for 2 min. Finally, the coverslips were mounted with DABCO.

### Measurement of insulin secretion

NIT-1 cells were washed with a modified Krebs-Ringer/bicarbonate-HEPES buffer (KRBH, 140 mmol/L NaCl, 3.6 mmol/L KCl, 0.5 mmol/L NaH_2_PO4, 0.5 mmol/L MgSO_4_, 1.5 mmol/L CaCl_2_, 2 mmol/L NaHCO_3_, 10 mmol/L HEPES, 0.1% BSA, pH7.4) and pre-equilibrated with DMEM containing 2.5 mmol/L glucose for 5 h at 37°C. Cells were then incubated for 35 min in KRBH buffer containing 2.5 mmol/L glucose (basal secretion) or KRBH buffer containing 20 mmol/L glucose (glucose-stimulated insulin secretion, GSIS). Incubates were collected and frozen for insulin assays [40], [41]. Insulin was determined by an ELISA kit (Linco) according to the manufacturer's protocol.

### siRNA transfection

The siRNAs targeting the mouse NOX2 or PTEN mRNA were transfected into NIT-1 cells using Tran Messenger TM Transfection Reagent (Qiagen) according to manufacture's instruction. A luciferase siRNA (FAM) was used as a negative control. RNAi oligonucleotides for transfection are 5′-UGCCAGAGUCGGGAUUUCU-3′dTdT for NOX2; 5′-GTATAGAGCGTGCAGATAA-3′dTdT for PTEN; 5′-UUCUCCGAACGUGUCACG -3′dTdT for FAM.

### Protein preparation of whole cell, nuclei, cytoplasm and mitochondrial fractions respectively

NIT-1 cells were lysed in a lysis buffer containing 50 mmol/l Tris-HCl (PH 8.0), 150 mol/l NaCl, 0.02% NaN3, 0.1% SDS, 1% NP-40, 100 µg/ml PMSF, 1 µg/ml aprotinin, 0.5% Sodium deoxycholate supplemented with a phosphatase inhibitor cocktail 1 and 2 (Sigma) and sonicated for 2 s to shear DNA. Cell lysates were centrifuged at 12,000 g for 10 min. Supernatant was used for Western blot analysis.

Proteins of nuclei and cytoplasm fraction in NIT-1 cells were prepared as previously described [35]. Briefly, the cells were collected and centrifuged for 20 s in a microcentrifuge followed by resuspending in Buffer 1 containing 10.0 mmol/L Hepes (pH 7.9), 10.0 mmol/L KCl, 1.5 mmol/L MgCl2 and 0.5 mmol/L dithiothreitol. After incubation at 4°C for 15 min, the cells were lysed by a Dounce homogenizer. The suspension was centrifuged for 20 s in a microcentrifuge. And subsequently, the supernatant (cytoplasm fraction) was collected and frozen. The pellet, which contained the nuclei, was resuspended in 150 µl Buffer 2 containing 20 mmol/L Hepes (pH 7.9), 20% v/v glycerol, 0.1 mol/L KCl, 0.2 mmol/L EDTA(PH 8.0), 0.5 mmol/L dithiothreitol and 0.5 mmol/L phenylmethanesulfonyl fluoride. Having been stirred at 4°C for 30 min, the nuclear extracts were centrifuged for 20 min at 4°C in a microcentrifuge. The supernatant was collected and stored at −80°C.

Proteins of mitochondrial in NIT-1 cells were prepared as previously described [42]. Briefly, the cells were collected and lysed on ice for 30 min in buffer A containing 20 mmol/L Hepes-KOH(PH 7.5), 10 mmol/L KCl, 1.5 mmol/L MgCl2, 1 mmol/L EGTA, 1 mmol/L EDTA (PH 8.0), 1 mmol/L dithiothreitol, 0.1 mmol/L PMSF, 1 µg/ml aprotinin and 250 mmol/L sucrose. After a consecutive centrifugation at 1,000 g for 5 min and 10,000 g for 15 min, the pellet, which contained mitochondrial fraction, was resuspended in buffer A and centrifuged at 100,000 g for 1 h. The supernatant was collected and stored at −80°C.

### Western blot analysis

Cell lysates (10–30 µg protein) were separated by 10% or 15% (for measuring insulin) SDS-PAGE, transferred to PVDF membrane (Millipore, pore size 0.20 µm), blocked with 5% nonfat dry milk for 60 min, and probed with antibodies at 4°C overnight. The blots were incubated with Horseradish peroxidase (HRP)-conjugated anti-IgG, followed by detection with ECL (Santa Cruz). Antibodies to p38MAPK, phosphor-p38MAPK, JNK, phosphor-JNK, Akt, phosphor-Akt, phosphor-p53, PTEN and phosphor-PTEN were purchased from Cell Signaling. Antibodies to I-κB, phosphor-I-κB, Bax, cytochrome C, HSP 70, p53, FOXO1, PDX-1, insulin and β-actin were obtained from Santa Cruz.

### Statistical analysis

All values are represented as means±SEM of the indicated number of measurements. One-way ANOVA test was used to determine significance, requiring p<0.05 for statistical significance.
